# Recent Advances in Understanding the Microbiology of the Female Reproductive Tract and the Causes of Premature Birth

**DOI:** 10.1155/2010/737425

**Published:** 2010-12-09

**Authors:** Xia Zhou, Rebecca M. Brotman, Pawel Gajer, Zaid Abdo, Ursel Schüette, Sam Ma, Jacques Ravel, Larry J. Forney

**Affiliations:** ^1^Initiative for Bioinformatics and Evolutionary Studies (IBEST), University of Idaho, Moscow, ID 83844-3051, USA; ^2^Department of Biological Sciences, Life Sciences South, Rm. 441A, University of Idaho, Moscow, ID 83844-3150, USA; ^3^Institute for Genome Sciences, University of Maryland School of Medicine, Baltimore, MD 21201-1559, USA; ^4^Department of Mathematics, University of Idaho, Moscow, ID 83844-3051, USA; ^5^Department of Statistics, University of Idaho, Moscow, ID 83844-3051, USA

## Abstract

Data derived from molecular microbiological investigations of the human vagina have led to the discovery of resident bacterial communities that exhibit marked differences in terms of species composition. All undergo dynamic changes that are likely due to intrinsic host and behavioral factors. Similar types of bacteria have been found in both amniotic fluid and the vagina, suggesting a potential route of colonization. Given that not all of the species involved in intrauterine infections are readily cultivated, it is important that culture-independent methods of analysis must be used to understand the etiology of these infections. Further research is needed to establish whether an ascending pathway from the vagina to the amniotic cavity enables the development of intrauterine infections.

## 1. Introduction

**Figure 1:**
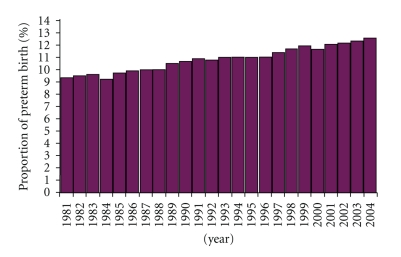
Percentage of all births classified as preterm in the USA, 1981–2004 (Source: Goldenberg et al. [16]).

Preterm birth is the leading cause of neonatal mortality worldwide [1], yet the underlying etiologies remain largely unknown. Despite the implementation of many public health measures and medical interventions, preterm births continue to increase (Figure 1). A strong body of evidence suggests that intrauterine infection is an important mechanism that might account for 25–40% of preterm births [2, 3]. However, this is probably a conservative estimate because many infections are likely to be subclinical, and the pathogens responsible for these infections may be difficult to detect with conventional culture techniques [4, 5]. Furthermore, our current understanding of normal vaginal microbiota, bacterial vaginosis, and the relationship to intrauterine infection and preterm birth is limited and incomplete. 

Efforts to understand the microbiology of the human vagina have been hampered in the past by the use of cultivation-based approaches that have significant limitations, and because longitudinal studies on the dynamics of vaginal bacterial communities have been lacking. Current efforts to understand the human microbiome and its role in preventing infections have entered the metagenomic era in which high throughput DNA sequencing technologies are used to characterize the diversity and function of microbial communities. Not only does this dramatically change the types of data routinely obtained from clinical samples [6], but it provides greater insight to microbial community structure, function, dynamics, and the interspecies interactions that are central to explaining how the human microbiota functions to maintain host health or predispose individuals to diseases [7–9]. We argue that in addition to the technical advances that these methodologies offer we need conceptual advances in the way these data are analyzed, interpreted, and translated into clinical practice. Through these advances our understanding of infectious processes and strategies to prevent and treat infections can be improved.

## 2. The Value of 16S rRNA Gene Sequence Analyses in Studies of the Human Microbiome

Our understanding of the composition of microbial communities associated with humans has been largely derived from studies that required the cultivation of microbial populations. Hence, our current understanding of microbe-host interactions is limited because the majority of microbial species resist cultivation in the laboratory [10]. Unquestionably, the cultivation of microorganisms is essential to fully understand the physiology and phenotypic properties of organisms, and thus invaluable to clinical microbiology. However, expansive studies done to assess inter- and intra-personal variation in microbial community composition or to explore ecological relationships and answer epidemiological questions require methods that provide detailed, in-depth information about microbial diversity while being cost-effective and amenable to high throughput sample analysis. In recent years, investigators have begun to rely on culture-independent methods based on analysis of 16S rRNA gene sequences to expand our knowledge of microbial diversity. These methods circumvent the need to cultivate organisms by analyzing nucleic acids directly extracted from samples. Typically the 16S rRNA genes in samples are amplified using primers that anneal to highly conserved regions of the gene and then amplicons are sequenced. Phylogenetic analysis of the sequences obtained allows for classification of the phylotypes (e.g., species) present and is a means to identify the numerically dominant species in communities, changes in community composition that occur in responses to treatments, the influences of habits and practices, and so on. As a result, this has become the favored approach to characterizing microbial populations and communities that reside in or on the human body [11–15]. 

It has been estimated that one must sample 80% of the species in the community to adequately assess microbial community diversity [17]. Thus, to adequately catalog the majority of microbial taxa in diverse communities, a large number of clones must be sequenced, which is both time-consuming and costly. This high “per sample” cost has historically limited the number of samples that can be analyzed, which in turn placed severe constraints on experimental designs. To alleviate this problem, Sogin et al. [18] pioneered the use of massively parallel DNA sequencing of short, hypervariable regions of 16S rRNA genes to produce detailed surveys of communities that include low abundance taxa. Using technology originally developed by 454 Life Sciences [19, 20] and now manufactured and distributed by Roche Applied Science over 1 million DNA sequence reads can be generated in a single pyrosequencing run, and sequence read lengths are approximately 400–500 bp. To simultaneously analyze multiple samples, each sample is amplified using primers that include a unique 6-bp sequence that provides a way to bin sequences during postsequencing data analysis. Upwards of 250 samples can be analyzed in a single sequencing run, yielding 4000 sequences per sample. By simply reducing the number of samples per run, the depth of coverage can be increased. This technology circumvents traditional approaches that require cloning, which allows users to avoid biases associated with library construction. Moreover, the novel sequencing chemistry permits the sequencing of regions of DNA that have secondary structure or unusual base composition. The lower cost and higher throughput of such technology when applied to 16S rRNA gene sequence analysis affords a way to sample microbial communities at depths that are orders of magnitude greater than is possible by traditional Sanger sequencing of cloned 16S rRNA amplicons [21].

## 3. Profiling Bacterial Diversity in the Human Vagina Based on the Analysis of 16S rRNA Gene Sequences

### 3.1. Bacterial Communities in the Human Vagina

In the past 100 years since the first microbiological study of the human vagina [22], lactobacilli have been thought to be the predominant members of normal postpubertal vaginal microflora [23]. Studies reliant on the cultivation of organisms have shown that a diverse array of other bacteria such as *Staphylococcus*, *Ureaplasma*, *Corynebacterium*, *Streptococcus*, *Peptostreptococcus*, *Gardnerella*, *Bacteroides*, *Mycoplasma*, *Enterococcus*, *Escherichia*, *Veillonella*, and *Bifidobacterium*, as well as the yeast *Candida* [24–26] can be present but typically in much lower numbers. The species of lactobacilli that have been cultivated from vaginal samples of healthy women and identified based on phenetic characters include *Lactobacillus jensenii*, *L. acidophilus*, *L. casei*, *L. gasseri*, *L. crispatus*, *L. plantarum*, *L. fermentum*, *L. cellobiosus*, *L. brevis*, *L. minutes,* and *L. salivarius* [27–29]. Few studies have been done to assess temporal variation in vaginal community composition within individuals, but those completed suggest that these communities are not subject to dramatic changes in healthy women, even during menses [30–32].

With advances in DNA sequencing technologies and decreased costs, our knowledge of human vaginal microbiota has greatly increased in recent years. Several studies have used culture-independent methods to characterize the vaginal microbial communities of reproductive-age, apparently healthy and asymptomatic women [33–38]. The analytical methodologies used and study designs have varied somewhat in terms of sampling different regions of the vagina, differences in the ethnic backgrounds of women sampled, the geographical location of populations, sampling times in relation to the menstrual cycle, and so on. Nonetheless, these studies are concordant in demonstrating that vaginal bacterial community composition differs both within and between individuals and several different kinds of communities are known to exist. Thus, a more complicated picture of vaginal microbiota in healthy, asymptomatic women has been painted. For example, in a previous study we analyzed 144 vaginal samples from White and Black women, a subset of those previously collected from more than 3,000 healthy women across North America [39]. The results showed that in 80% of the women microorganisms phylogenetically related to *Lactobacillus iners*, *L. crispatus*, *L. jensenii*, or *L. gasseri* dominated sampled vaginal communities. Overall, *L. iners* was the most common species of *Lactobacillus* in women of both ethnic groups having been recovered in 66% of the women sampled. *L. iners* is an underappreciated member of the normal vaginal biota, as it does not grow on Rogosa agar that is typically used to isolate lactobacilli. The remainder of communities had low numbers of lactobacilli, exhibited greater species evenness, and included high numbers of clones most closely related to *Atopobium* and genera of the order *Clostridiales*, including *Megasphaera*, *Dialister*, *Anaerococcus*, *Finegoldia*, *Peptostreptococcus*, and *Eubacterium*. In addition, 20–30% of the clones from these communities were from novel clades in the phylum Firmicutes. Comparable results were obtained in a recent study of healthy, reproductive-age Japanese women [40]. The findings of these studies indicate there are a limited number of different kinds of vaginal microbial communities in asymptomatic, apparently healthy women. Moreover, from studies of adolescent women (13–15 y) [41], it appears that these communities are established in puberty and may reside in women until menopause. 

Recently, we completed a more detailed and expansive study to characterize vaginal microbiota using high-throughput methods based on pyrosequencing of barcoded 16S rRNA genes [42]. The subjects were a cohort of 396 North America asymptomatic women equally representing four ethnic backgrounds (Asian, White, Black, and Hispanic). Women were recruited at three clinical sites: two in Baltimore at the University of Maryland School of Medicine and one in Atlanta, at Emory University. The participants self-identified their race. All women enrolled in the study were not pregnant, of reproductive age ranging from 12 to 45 years (mean 30.6 ± 7.32 years), had regular menstrual cycles (25–35-day menstrual cycles), with a history of sexual activity, and had not taken any antibiotic or antimycotic compounds in the past 30 days. Women were asked to refrain from sexual activity in the 48 h before the visit. The vaginal swabs were self-collected by women who were not menstruating or using contraceptive devices, such as NuvaRing [42]. In total there were 282 phylotypes identified in these women. The communities clustered into five groups; four of which were dominated by *Lactobacillus iners*, *L. crispatus*, *L. gasseri,* or *L. jensenii*, while the fifth had lower proportions of lactic acid bacteria and higher proportions of strict and facultative anaerobes. This low-*Lactobacillus* group accounted for about 25% of the women sampled. Aside from the different *Lactobacillus* species, the most abundant taxa identified in the human vagina were *Prevotella*, *Megasphaera*, *Sneathia*, *Atopobium*, *Streptococcus*, *Dialister*, *Lachnospira*, *Anaerococcus*, *Peptoniphilus*, *Eggerthella*, *Finegoldia*, *Rhodobaca*, *Anaerotruncus*, *Ureaplasma*, *Mycoplasma*, *Aerococcus*, *Parvimonas*, *Staphylococcus*, *Corynebacterium*, *Veillonella*, *Gardnerella*, *Gemella*, and *Mobiluncus*. The most commonly observed taxa in each community group are shown in Table 1. The results further showed that high bacterial species diversity was observed in all vaginal communities, even those where the phylotype abundance distribution was highly skewed and dominated by one or a very few phylotypes. 

**Figure 2:**
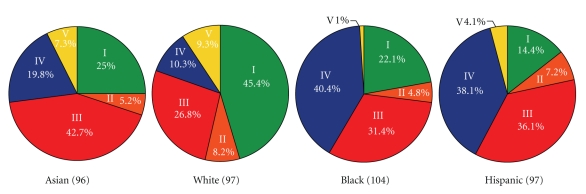
Proportions of community groups found in women of different ethnic groups (Source: [42]).

The study cohort consisted of roughly equal numbers of four ethnicities (white, Asian, black, and Hispanic), and this offered the opportunity to assess the relationship of ethnic background on vaginal bacterial community composition. The proportions of each community group varied among the four ethnic groups (Figure 2), and these differences were statistically significant [*χ* ^2^(10) = 36.8, *P* < .0001]. No statistically significant associations were observed between age and community types within or across ethnic groups. Vaginal bacterial communities dominated by species of *Lactobacillus* (groups I, II, III, and V) were found in 80.2% and 89.7% of Asian and white women, respectively, but in only 59.6% and 61.9% of Hispanic and black women, respectively. We found that community group IV was overrepresented in Hispanic (34.3%) and black (38.9%) women as compared with Asian (17.6%) and white (9.3%) women. From these data we conclude that vaginal bacterial communities not dominated by species of *Lactobacillus* are common and appear frequently in black and Hispanic women. The data from this study are in accordance with the results of Zhou et al. [37, 39, 40], who studied the vaginal bacterial communities of white, black, and Japanese women. 

### 3.2. Temporal Dynamics of Vaginal Bacterial Communities

Most studies of vaginal microbiology have employed a cross-sectional study design in which individuals are sampled at one discrete time point or used an interval-censored study design such that participant samples are obtained every few weeks or months [30–32, 43]. As a result little is known about the temporal dynamics of vaginal bacterial communities, and many have the mistaken impression that the composition of these communities is comparatively invariant over time, except perhaps during menstruation and following other deliberate disturbances such as sexual activity or vaginal douching. However, as our understanding of the human microbiome improves, it is becoming increasingly apparent that the bacterial communities of some habitats can markedly change over time and in response to environmental changes. For example, differences or changes in diet can have profound effects on the composition of bacterial communities of the gastrointestinal tract [44, 45]. To understand how microbial communities in the human body fluctuate in response to either defined events or stochastic processes, dynamic community profiling studies are needed [6]. 

Recently, we completed the sequencing of archived specimens from a longitudinal study of 33 reproductive age women who self-collected vaginal swabs every 3 days over a 16-week time period (see [46], Gajer et al. unpublished). The vaginal bacterial communities of nearly all women were dynamic and exhibited marked changes in the relative abundances of species over time. Usually these shifts involved changes in the relative proportions of species, but in some cases a distinct turnover in species composition occurred that persisted over time and was akin to an alternative equilibrium state. Factors that influence the dynamics of communities are currently under investigation, but may include hormonal fluctuations, sex practices, frequency of sex, use of vaginal douches, and other feminine hygiene products, or other factors.

## 4. The Enigma of Bacterial Vaginosis

The risk of preterm birth and low birth weight infants is markedly increased in women with bacterial vaginosis [47, 48], yet the etiology of bacterial vaginosis remains an enigma [49]. In simple terms, bacterial vaginosis is said to reflect a disturbed vaginal ecosystem in which *Lactobacillus* species are reduced in number and the community is “overgrown” by strictly anaerobic organisms [50, 51]. Clinically, the diagnosis of bacterial vaginosis requires three of the following four symptoms or signs [52]: (a) homogeneous, thin, white discharge that smoothly coats the vaginal walls, (b) presence of clue cells on microscopic examination, (c) pH of vaginal fluid >4.5, and (d) a fishy odor of vaginal discharge before or after addition of 10% KOH. Alternatively, bacterial vaginosis is diagnosed based on the assessment of bacterial cellular morphologies observed in samples using criteria first introduced by Spiegel et al. [53] and then modified by Nugent et al. [54]. The diagnosis of bacterial vaginosis using the Nugent criteria is based on a numerical scoring system (0–10). The score reflects the relative abundances of three kinds of bacterial cell morphotypes in Gram-stained vaginal smears, namely, large gram-positive rods (*Lactobacillus*), small gram-variable rods (*G. vaginalis*/*Bacteroides* spp.), and curved gram-variable rods (*Mobiluncus*). 

The diagnostic criteria used are a critical issue in studies on the etiology of bacterial vaginosis. While numerous studies have shown that women with high numbers of *Lactobacillus* species generally do not have bacterial vaginosis, it is a logical fallacy to conclude that women whose vaginal communities have few or no *Lactobacillus* species have bacterial vaginosis. Unfortunately, this fallacy is the premise of the Nugent criteria wherein the degree of “healthiness” is largely influenced by scoring the relative abundance of *Lactobacillus* species with typical cell morphology. We assert that while “normal and healthy” can be equated with high numbers of lactobacilli, the converse—that “unhealthy” can be equated with low numbers of or no lactobacilli—is not necessarily true [55]. We postulate that, because of this logical fallacy, bacterial vaginosis is often over-diagnosed by Gram's staining. This could partly account for the reported high incidence of so-called asymptomatic bacterial vaginosis in reproductive-age women [56, 57] and could also explain a proportion of bacterial vaginosis treatment failures and apparent recurrences of bacterial vaginosis in women [58, 59]. 

This does not deny the fact that vaginal communities of women with symptoms of bacterial vaginosis have high numbers of strictly anaerobic bacteria, many of which are various taxa that belong to the order *Clostridiales*. Several studies have reported this to be the case [60, 61]. We postulate that the presence of these organisms in high number is necessary but not sufficient to elicit the symptoms associated with bacterial vaginosis, and that differences in the complex of symptoms that become manifest are likely dictated by differences in the immune response of a host. This seems sensible given that disease results not only from the ill effects of microbial activities and products, but also from the nature and severity of the host immune response to the organism(s). This is apparent if one considers that the clinical diagnosis of infection depends upon the identification of the four signs of inflammation: dolor (pain), rubor (redness), calor (heat), and tumor (swelling) all of which reflect host inflammatory responses (http://www.aboutinflammation.com/fourclassicsymptomsofinflammation.html).Thus, it is logical to suggest that when examining the vaginal habitat, clinicians might also focus on the microbe-host immune system interaction [62]. Yet, the current diagnosis of asymptomatic bacterial vaginosis relies only on the microbial component of this equation and ignores the host component. Thus it seems unreasonable that the diagnosis of bacterial vaginosis should be based solely on the absence of certain taxa (lactobacilli) and presence of others (strict anaerobes). A similar dilemma occurs in clinical medicine when asymptomatic patients present with bacteria in their urine. For women, a diagnostic criterion for asymptomatic bacteriuria is two consecutive midstream clean-catch urine specimens with isolation of the same species in quantitative counts of at least 100,000 CFUs per mL of urine [63]. And in the case of asymptomatic bacteruria, there are only a few clinical circumstances in which antibiotic treatment has been shown to benefit the patient. 

It is important to note in the context of asymptomatic bacterial vaginosis that studies suggest that women with low-*Lactobacillus* dominated microbiota (many of which would be classified as asymptomatic bacterial vaginosis) are at greater risk for adverse outcomes including STD/HIV infection upon exposure and poor obstetric outcomes [64, 65].

### 4.1. Normal Vaginal Microbiota

The results of studies done using cultivation-independent methods require that we revise our perceptions of the bacterial species found in the vaginas of normal and healthy women. As mentioned above, recent work by Ravel et al. [42] showed that vaginal bacterial communities could be clustered into five groups demonstrating that there is no single core microbiome. These groups can be readily distinguished on the basis of two criteria: (a) whether the constituent communities are dominated by *Lactobacillus* or not and (b) the particular species of *Lactobacillus* present. In the past it has been claimed that the vaginal bacterial communities of healthy women are dominated by species of *Lactobacillus* that produce hydrogen peroxide [49, 51, 66]. This appears to be true for some but not all women. The most common communities are dominated by *L. iners*, a species that can be characterized by the inability to produce hydrogen peroxide [23]. Moreover, not only does this organism resist cultivation on commonly used media (which probably accounts for its absence from most surveys done in the past that relied on cultivation), but the cell morphology is atypical by being about 50% smaller (length and width) than the other species of *Lactobacillus* common to the human vagina (Yuan and Forney, unpublished). This could confound the diagnosis of bacterial vaginosis based on Nugent criteria. The fifth group of vaginal communities found in asymptomatic women is heterogeneous in terms of species composition and typified by a dearth of lactobacilli and higher proportions of strictly anaerobic bacteria including *Prevotella*, *Dialister*, *Atopobium*, *Gardnerella*, *Megasphaera*, *Peptoniphilus*, *Sneathia*, *Eggerthella*, *Aerococcus*, *Finegoldia*, and *Mobiluncus*. A large proportion (27%) of White, Black, Hispanic, and Asian women in North America have vaginal communities that cluster within this group, and they are particularly frequent in Hispanic and Black women (38 and 40%, resp.; Figure 2). The fact that these communities are not dominated by species of *Lactobacillus* has led some to presume that these women have bacterial vaginosis [50, 56]. We postulate that these asymptomatic women with vaginal communities lacking appreciable numbers of lactobacilli may be misdiagnosed as having bacterial vaginosis if Nugent criteria are used. The fact that these communities appear to reflect a natural state and not disease might account for the high recurrence rates and spontaneous cure rate for asymptomatic bacterial vaginosis that have been observed [58, 59, 67, 68]. If this is the case, then vaginal bacterial communities that lack lactobacilli may simply represent another difference found among individuals and highlight the importance of personalized medicine wherein differences among individuals are respected. 

There is a widespread discussion over whether asymptomatic gynecologic patients with bacterial vaginosis should be treated [69] since the risk of therapy must be weighed against the benefit to patients, and there is increasing awareness of the need to restrict antibiotic use so as to avoid selection for antibiotic resistance [70, 71]. The arguments presented above suggest that the use of antibiotics for the treatment of asymptomatic bacterial vaginosis might not be sensible since disturbance of a natural state is the “cure” one would be attempting to affect. It should be noted that these communities may tend to revert to their original state once antibiotic therapy has been completed, and this could well account for a portion of the so-called treatment failures that are observed in trials done to assess the efficacy of antibiotic therapy for curing bacterial vaginosis [58, 72].

### 4.2. Treatment of Asymptomatic Bacterial Vaginosis in Pregnant Women

Controversy surrounds whether pregnant women should be screened for the occurrence of asymptomatic bacterial vaginosis and treated with antimicrobial agents to prevent preterm birth. The basic rationale for screening and treatment is that bacterial vaginosis is associated with intra-amniotic infection and therefore is considered a risk factor for preterm delivery. However, evidence compiled in the Cochrane Reviews does not support the concept of widespread screening for bacterial vaginosis and treatment to prevent premature delivery [73–75]. It is important to note that a history of a prior preterm birth is the most significant clinical factor in identifying women with a propensity for preterm labor and delivery [76, 77]. The Centers for the Disease Control, as well as prominent leaders in the field of infectious disease in obstetrics and gynecology, have recommended that only patients at high risk for preterm delivery—specifically only those with a previous history of a spontaneous preterm birth—should be treated with antibiotics if they are found to have bacterial vaginosis [62].

## 5. Intrauterine Infection and Preterm Birth

Intrauterine infections are a frequent and important mechanism leading to preterm birth. Intrauterine infection begins in the decidua (uterine lining), extends to the space between the amnion and chorion, and finally reaches the amniotic cavity and fetus [2]. Bacteria have been cultured from the chorioamnion in 15% of nonlaboring women with intact membranes who are undergoing caesarean delivery [2]. Likewise, half of all placentas delivered before the end of the second trimester have been shown to harbor bacteria in the chorion as detected by culture [78]. The prevalence of infection is found to be even higher when molecular methods are used to detect bacteria. When fluorescence *in-situ* hybridization is done using a DNA probe specific for a conserved region of the bacterial 16S rRNA gene, then bacteria are found in the membranes of up to 70% of women undergoing elective caesarean section at term [79]. Since these were not cases of preterm birth, these findings suggest that the presence of bacteria in the chorioamnion alone is not always sufficient to cause an inflammatory response that leads to preterm labor and preterm birth. In contrast, an inflammatory response is observed in the amniotic fluid of more than 80% women in early preterm labor with intact membranes. Based on these data, it seems there are two conditions essential for intrauterine infections to cause preterm birth. First, the infectious organisms must enter the amniotic cavity and be recognized as foreign by the host immune system. Second, the bacterial numbers must breach some threshold to trigger an intra-amniotic inflammatory response, which in turn induces preterm labor [80].

### 5.1. Bacterial Species Found in Amniotic Fluid

*Ureaplasma urealyticum*, *Fusobacterium* sp., and *Mycoplasma hominis* are the bacterial species most commonly isolated from the amniotic cavity of women with preterm labor and intact membranes [81, 82]. Other microorganisms found in the amniotic fluid include *Streptococcus agalactiae*, *Peptostreptococcus* sp., *Staphylococcus aureus*, *Gardenerella vaginalis*, *Streptococcus viridians,* and *Bacteroides* sp. Occasionally, *Lactobacillus* sp., *Escherichia coli*, *Enterococcus faecalis*, *Neisseria gonorrhea,* and *Peptococcus* sp., while *Haemophilus influenzae*, *Capnocytophaga* sp., *Stomatococcus* sp., and *Clostridium* sp. are rarely recovered [83–85]. More than one microorganism is isolated from 50% of patients in which the amniotic cavity is infected [79]. 

**Figure 3:**
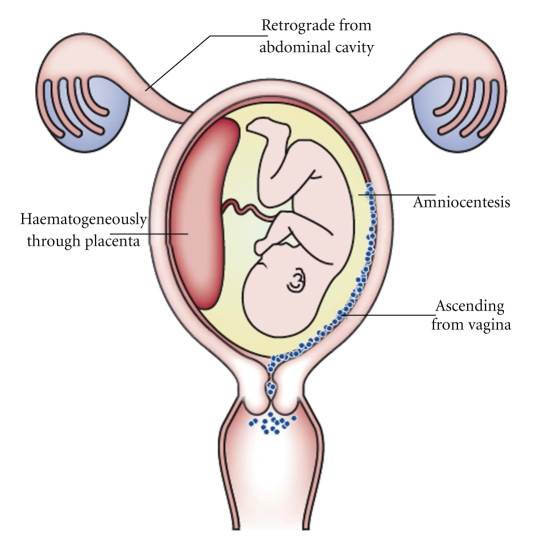
Potential routes of intrauterine infection (Source: Goldenberg et al. [16]).

In a recent, very comprehensive analysis, DiGiulio and colleagues characterized bacterial 16S rRNA gene sequences in amniotic fluid from women in preterm labor [5]. They found 18 taxa in the amniotic fluids using molecular methods of analysis while only 11 taxa were recovered using culturing methods. In addition, 9 samples were positive only by PCR amplification of 16S rRNA genes, indicating that false negative results can be obtained by using only cultivation methods. In this study, *Mycoplasma* sp., *Ureaplasma* sp., *Streptococcus* sp., *Lactobacillus* sp., *Prevotella* sp., *Delftia* sp., *Neisseria* sp., *Fusobacterium* sp., *Sneathia* sp., and *Leptotrichia* sp. were found in amniotic fluids. In another study by Han et al. [86], twice the numbers of bacterial taxa were identified in the amniotic fluids of preterm delivery patients using cultivation-independent methods as compared to cultivation-dependent methods. Most taxa were similar to those of DiGiulio's study, but in addition *Shigella* sp., *Bacteroides* sp., *Bergeyella* sp., and *Peptostreptococcus* sp. were observed. While some bacteria in amniotic fluid have been associated with skin, fecal, and gut microbiota, most are related to those found in the human vagina. This suggests a potential connection between the bacterial species in amniotic fluid with those in the vagina, with the latter being a potential source of infecting organisms [16] (see Figure 3).

## Figures and Tables

**Table 1:** Most commonly observed taxa in the vaginal bacterial community groups defined by Ravel et al. [42].

Group I (N* = 105)			Group II (*N* = 25)			Group III (*N* = 135)			Group IV (*N* = 108)			Group V (*N* = 21)	

Taxa	Percentage of samples		Taxa	Percentage of samples		Taxa	Percentage of samples		Taxa	Percentage of samples		Taxa	Percentage of samples
*L.crispatus*	100.0		*L.gasseri*	100.0		*L.iners*	100.0		*Prevotella*	99.1		*L.jensenii*	100.0
*Lactobacillales.6*	99.1		*Anaerococcus*	84.0		*Lactobacillales.2*	97.8		*Dialister*	93.5		*Lactobacillales.5*	95.2
*L.iners*	76.2		*Lactobacillales.1*	84.0		*Lactobacillales.5*	97.0		*Peptoniphilus*	88.9		*L.iners*	66.7
*Lactobacillales.5*	71.4		*Peptoniphilus*	76.0		*Prevotella*	56.3		*Anaerococcus*	80.6		*Prevotella*	61.9
*L.jensenii*	69.5		*Prevotella*	72.0		*L.crispatus*	53.3		*Atopobium*	79.6		*Finegoldia*	61.9
*L.vaginalis*	64.8		*Dialister*	68.0		*L.jensenii*	51.1		*L.iners*	78.7		*Corynebacterium*	61.9
*Prevotella*	53.3		*L.iners*	60.0		*Ureaplasma*	48.9		*Gardnerella*	78.7		*L.crispatus*	57.1
*L.gasseri*	49.5		*Finegoldia*	60.0		*Dialister*	40.0		*Megasphaera*	76.9		*L.gasseri*	52.4
*Anaerococcus*	41.9		*Streptococcus*	56.0		*Finegoldia*	39.3		*Sneathia*	70.4		*Lactobacillales.7*	52.4
*Finegoldia*	41.0		*Atopobium*	52.0		*L.gasseri*	38.5		*Eggerthella*	70.4		*Streptococcus*	47.6
*Corynebacterium*	37.1		*Corynebacterium*	52.0		*Corynebacterium*	38.5		*Parvimonas*	70.4		*Anaerococcus*	42.9
*Ureaplasma*	33.3		*L.vaginalis*	48.0		*Anaerococcus*	36.3		*Finegoldia*	67.6		*Peptoniphilus*	42.9
*Lactobacillales.2*	32.4		*Staphylococcus*	44.0		*L.vaginalis*	34.8		*Ruminococcaceae.3*	67.6		*Dialister*	38.1
*Peptoniphilus*	31.4		*Gardnerella*	44.0		*Peptoniphilus*	34.1		*Prevotellaceae.2*	67.6		*Staphylococcus*	33.3
*Staphylococcus*	29.5		*L.crispatus*	36.0		*Streptococcus*	31.1		*Aerococcus*	63.0		*L.vaginalis*	33.3
*Streptococcus*	28.6		*Lactobacillus.1*	36.0		*Staphylococcus*	31.1		*Mobiluncus*	61.1		*Ureaplasma*	28.6
*Dialister*	24.8		*Lactobacillales.5*	36.0		*Atopobium*	25.2		*Anaeroglobus*	59.3		*Gardnerella*	28.6
*Lactobacillus.2*	23.8		*Ureaplasma*	32.0		*Aerococcus*	23.7		*Porphyromonas*	56.5		*Lactobacillales.2*	28.6
*Atopobium*	21.0		*Actinomyces*	32.0		*Sneathia*	23.0		*Gemella*	54.6		*Propionibacterium*	28.6
*Megasphaera*	20.0		*L.jensenii*	28.0		*Megasphaera*	20.7		*L.crispatus*	51.9		*Atopobium*	19.0
*Exiguobacterium*	17.1		*Bacteroides*	24.0		*Lactobacillales.7*	18.5		*Corynebacterium*	51.9		*Clostridiales.17*	19.0
*Clostridium*	14.3		*Clostridiales.17*	24.0		*Veillonella*	16.3		*Ruminococcaceae*	50.9		*Gemella*	19.0
*Clostridiales.17*	13.3		*Campylobacter*	24.0		*Peptostreptococcus*	16.3		*Peptostreptococcus*	48.2		*Varibaculum*	19.0
*Lactobacillus.4*	13.3		*Lachnospiraceae.7*	24.0		*Gemella*	15.6		*Streptococcus*	46.3		*Megasphaera*	14.3
*Bacteroides*	12.4		*Bifidobacterium*	24.0		*Lactobacillales.1*	15.6		*Ruminococcaceae.4*	46.3		*Bacteroides*	14.3
*Aerococcus*	10.5		*Parvimonas*	20.0		*Gardnerella*	14.8		*Prevotellaceae.1*	45.4		*Peptostreptococcus*	14.3
*Lactococcus*	10.5		*Porphyromonas*	20.0		*Lactobacillus.2*	14.1		*Ureaplasma*	39.8		*Lachnospiraceae.7*	14.3
*Sneathia*	9.5		*Varibaculum*	20.0		*Clostridiales.17*	12.6		*Clostridiales.17*	37.0		*Actinomyces*	14.3
*Parvimonas*	9.5		*Exiguobacterium*	20.0		*Porphyromonas*	11.9		*Segniliparus*	37.0		Coriobacteriaceae.1	14.3

Total number of taxa observed in all samples within a group	174			125			169			232			105

*Total number of subjects within a group.
